# The Use of Constituent Spectra and Weighting in Extended Multiplicative Signal Correction in Infrared Spectroscopy

**DOI:** 10.3390/molecules27061900

**Published:** 2022-03-15

**Authors:** Johanne Heitmann Solheim, Boris Zimmermann, Valeria Tafintseva, Simona Dzurendová, Volha Shapaval, Achim Kohler

**Affiliations:** Faculty of Science and Technology, Norwegian University of Life Sciences, Drøbakveien 31, 1432 Ås, Norway; boris.zimmermann@nmbu.no (B.Z.); valeria.tafintseva@nmbu.no (V.T.); simona.dzurendova@nmbu.no (S.D.); volha.shapaval@nmbu.no (V.S.); achim.kohler@nmbu.no (A.K.)

**Keywords:** preprocessing, extended multiplicative signal correction, infrared spectroscopy

## Abstract

Extended multiplicative signal correction (EMSC) is a widely used preprocessing technique in infrared spectroscopy. EMSC is a model-based method favored for its flexibility and versatility. The model can be extended by adding constituent spectra to explicitly model-known analytes or interferents. This paper addresses the use of constituent spectra and demonstrates common pitfalls. It clarifies the difference between analyte and interferent spectra, and the importance of orthogonality between model spectra. Different normalization approaches are discussed, and the importance of weighting in the EMSC is demonstrated. The paper illustrates how constituent analyte spectra can be estimated, and how they can be used to extract additional information from spectral features. It is shown that the EMSC parameters can be used in both regression tasks and segmentation tasks.

## 1. Introduction

Infrared spectroscopy has over the past decades grown to become one of the most prominent techniques for non-destructive biochemical characterization of biological materials. An ideal absorbance spectrum is approximately proportional to the concentrations of the absorbing compounds in the material. However, physical effects, which are caused by the measurement setup or the sample morphology, may interfere with the absorbance and disturb the signal. This leads to scaling effects in the spectra, as well as baseline shifts and deformations. Preprocessing methods are therefore needed to clean the data prior to the interpretation [1]. By removing interfering effects from the absorbance spectra, less data is needed to calibrate multivariate models or machine learning models [2]. Several preprocessing methods have been proposed in literature. By taking the first or second derivative of the spectra, broad baseline distortions are suppressed [3]. They can also be suppressed by rubber band correction. Scaling variations can be handled with peak normalization or vector normalization [4]. All these methods are filtering methods, which means that they simply remove the unwanted effects in the spectra.

After multiplicative signal correction (MSC) was introduced in 1983 by Martens et al. [5], MSC and its extended version EMSC (extended MSC) has been favored for its flexibility and versatility. EMSC is a model-based technique, which means that it builds on a physical model of scattering and absorption. It builds on the Beer–Lambert law, and can be extended by adding the model spectra of known physical or chemical variations, called the constituent spectra [6]. Instead of simply removing unwanted effects, the effects are explicitly modeled and parameterized before they are removed. The EMSC parameters are thus available for the analysis. This is beneficial since physical effects can be related to the sample morphology, and therefore may carry relevant information (see Figure 11 in [7,8,9]).

In the EMSC, we distinguish between unwanted features in the measured spectra, which should be removed (interferents), and features which should not be removed (analytes). Typical interferent signals can be scattering features, e.g., Mie scattering or interference fringes, or chemical signals from paraffin embedding [10]. Analyte spectra are known chemical variations, e.g., a known chemical compound in the sample.

While the literature suggests the use of constituent spectra in EMSC [11,12], it is not clear how one or several constituent spectra can be defined such that confounding with the reference spectrum is avoided. This has created some uncertainty in the use of constituent spectra in EMSC in the infrared community (private communications). The paper at hand therefore aims to develop some clear strategies for how to create and use constituent spectra in the EMSC and to discuss major pitfalls when using constituent spectra. We show how analyte constituent spectra can be estimated from the data, and how they can be used for predicting the proportion of a known constituent, or to segment infrared spectroscopic images. Further, we illustrate the effect of using different normalization techniques, and show how normalization may introduce artifacts in the spectra. The use of weights in the EMSC normalization is also discussed, and we show that the optimal normalization approach depends on the aim of the analysis. We present three case studies. In case study I, we discuss different normalization strategies, and in case studies II and III we discuss the use of constituent spectra.

## 2. Results and Discussion

### 2.1. The Effect of Different Normalization Procedures and the Importance of Weighting in the EMSC

Preprocessing methods in vibrational spectroscopy can be divided into filtering methods and model-based methods. Common filtering methods include vector normalization, peak normalization, and derivatives. Model-based methods, such as EMSC, are often preferred to filtering methods since they explicitly model and parameterize physical and chemical variation. Information is therefore not lost during preprocessing. In addition, model-based methods are recognized for their flexibility in modeling specific chemical or physical variations. The EMSC model is described in Section 3.2.

In the following, we demonstrate how normalization is achieved by EMSC, and compare it to vector normalization and peak normalization. One should note that both vector normalization and peak normalization require the spectra to be baseline corrected, while EMSC performs baseline correction and normalization simultaneously.

Peak normalization ensures that the absorbance value of the selected peak is equal for all the spectra. The physical interpretation of peak normalization is that one standardizes the sample biomass to the amount of one compound, e.g., proteins (through the amide I peak at 1653 cm${}^{-1}$). Vector normalization, on the other hand, normalizes according to the area under the curve, which is the total absorbance. We will show that by applying weights in the EMSC, similar results as for vector normalization can be achieved.

**Figure 1:**
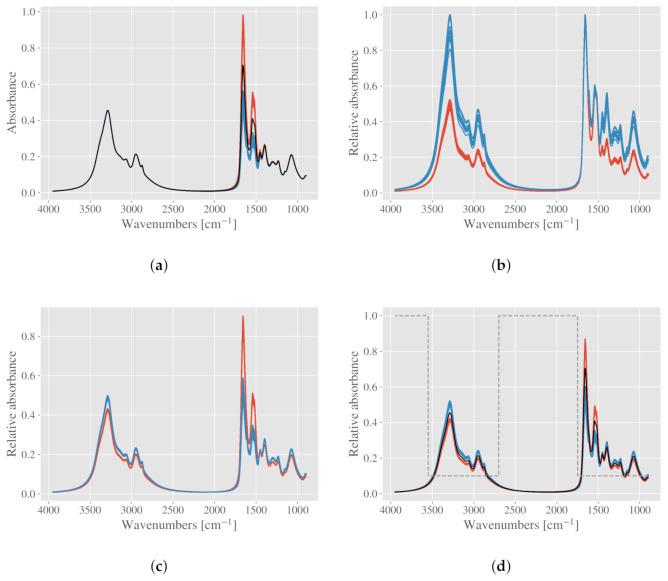
Different normalization procedures demonstrated on a simulated dataset. (**a**) Simulated spectra showing the two chemically different groups in different color. The groups are simulated with chemical differences in the amide I and II region. The mean spectrum is shown in black. (**b**) Peak normalization according to the amide I peak. (**c**) Vector normalization. (**d**) Weighted EMSC normalization is shown with weights in dashed gray. The mean spectrum in black is used as reference. Only a constant baseline was included in the EMSC (MSC).

To illustrate the effect of different normalization procedures, we have simulated a dataset consisting of two chemically different groups. A template spectrum was created by fitting Lorentzian functions to a Matrigel spectrum [13], and subsequently the peak heights of the Lorentzian functions were adjusted systematically to create the two groups. Only the amide I and II peaks were changed such that the peak heights for the first group were scaled between 0.85 and 1 compared to the original template, while for the second group they were scaled between 0.4 and 0.55. There are in total 20 spectra per group. The simulated spectra are shown in Figure 1a, and we refer to the groups as “red” and “blue” according to this figure. The mean of all spectra is shown in black. It is evident from the figure that these spectra have no need for baseline correction, and we can therefore perform peak normalization or vector normalization directly. Peak normalization according to amide I is shown in Figure 1b, while vector normalization is shown in Figure 1c. In the latter, the spectra are vector normalized according to the mean spectrum of the two groups. With vector normalization, the spectra are standardized to the total absorbance, which can be considered as an equivalent for the total biomass that is transmitted by infrared radiation during the absorption measurement. This means that in this case, chemical differences are present in all spectral regions. This is reasonable, since a decrease in amide I and II concentration necessarily means that the concentration of other compounds must increase. For peak normalization, the concentration differences are even more pronounced, since they are standardized to one compound rather than the whole biomass. It is assumed here that the amide I component is equal for all samples in a group.

In order to assign more or less importance to different spectral regions in the normalization process, we perform a weighted EMSC. Applying weights means that the model spectra are all multiplied with the weight spectrum prior to the least squares regression performed in the EMSC modeling. Therefore, weighting in the EMSC is not the same as a weighted least squares regression. Weighting is a powerful tool when normalizing spectra with EMSC. Weights are applied to regions where the variation is expected to be high also after the normalization. There is no universal strategy for applying weights, and before applying weights, one should decide what kind of normalization one seeks. If one expects chemical differences to be significant, while also expecting no variations in the baseline, one could apply low weights to the chemically active regions. Doing this for the two simulated groups, one obtains a similar result as for vector normalization, as shown in Figure 1d. The spectra were corrected with an EMSC with only a constant baseline, with the mean of the two groups, shown in black, used as a reference. The weights used in the EMSC are shown in a grey dashed line.

It should also be pointed out that EMSC normalization with weighting in many cases outperforms vector normalization and peak normalization. For example, for spectra that exhibit strong baseline variations due to scattering, which is hard to remove completely, vector normalization and peak normalization are ineffective normalization methods. In these cases, EMSC can be used in combination with weights that put less priority to normalization of the baseline. An example is strong baseline variations in high throughput screening (HTS) FTIR spectra of biological particles. Scattering is strongly connected to the size of the particles, and strong variations in the baseline can therefore be observed for samples of varying particle size [14]. By down-weighing the chemically inactive regions in the EMSC, less priority is put to the varying baseline.

It is worth mentioning that there is no universal strategy for preprocessing infrared spectra and the optimal strategy depends on the goal of the analysis. Often it is desired to obtain spectra that can be interpreted visually and with multivariate methods. This may be the case when using unsupervised or supervised methods. However, when the goal is to achieve the best performance of a supervised model, visual interpretability may not be the first priority and a different preprocessing strategy may be chosen. It is common practice when optimizing the performance of multivariate models to evaluate different preprocessing techniques in the calibration process, and select the best performing strategy [3]. We want to refer to our recently published study [2] where we compare a large range of different preprocessing techniques, chemometrics, and machine learning classifiers for different spectroscopic datasets.

### 2.2. The Effect of Different Normalization Procedures—Case Study I

In the first case study, different normalization procedures are applied to the oleaginous filamentous fungi dataset. The dataset is described in Section 3.1.1. We normalize according to either total biomass, or to the total lipid content. It follows that the optimal normalization procedure depends on the aim of the subsequent analysis. We compare the two normalization procedures, EMSC normalization according to total biomass or total lipid content.

Spectra of oleaginous filamentous fungi were calibrated against the total percentage of fat in the biomass, or the concentration of polyunsaturated fatty acids (PUFA) relative to the total fat percentage, by two separate partial least squares regression (PLSR) models. We will investigate how different normalization methods for EMSC affects the two PLSR models. Not surprisingly, the optimal normalization strategy differs for the two regression models.

**Figure 2:**
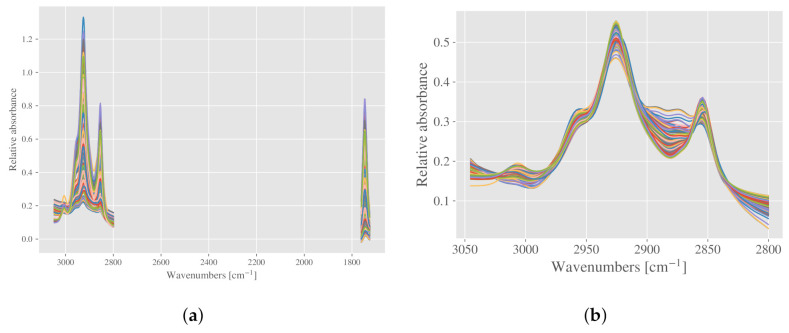
Different normalization strategies demonstrated on spectra of filamentous fungi. (**a**) Strategy A: the full spectra are first normalized with EMSC (described in the text), before the lipid regions are selected. (**b**) Strategy B: the lipid region at 2800–3590 cm${}^{-1}$ is selected prior to EMSC normalization.

The first normalization strategy, which we refer to as strategy A, is to first normalize the full spectra by a basic EMSC, before selecting the lipid regions. Normalization with EMSC is done on the full spectra to normalize according to the total biomass. After normalizing according to biomass, the lipid regions 1765–1727 cm${}^{-1}$ and 3050–2800 cm${}^{-1}$ are selected as input for both PLSR models; predicting total fat and PUFA. The normalized spectra are shown in Figure 2a.

The second strategy, which we refer to as strategy B, is to select the lipid region 3050–2800 cm${}^{-1}$, before performing an EMSC with polynomials up to 1st degree. With this procedure, the spectra are roughly standardized according to the lipid content. The reason for omitting the ester peak at 1765–1727 cm${}^{-1}$ is described in the following. Since each of these regions are very narrow, but together they span a quite big spectral range (3050–1727 cm${}^{-1}$), the preprocessing needs to be done separately to avoid creating extra baseline variations (see Supplementary Materials for more details). When the regions are preprocessed separately, the ester peak becomes very similar for all spectra, losing most of its predictive value. The peak still retains some predictive value for total lipid content, however the effect is small (results not shown). The normalized spectra using only the region 3050–2800 cm${}^{-1}$ are shown in Figure 2b, and they are used as input for both PLSR models.

Training and testing was performed as described in the following. The first set of biological replicates serve as the training set, while the second as the test set. All PLSR models were built by first optimizing the number of components ($A_{opt}$) in a 6-fold cross-validation procedure, where 70% of the samples served as the calibration set and the rest as validation within the fold. The samples were shuffled before splitting, but technical replicates were not split across calibration and validation sets. The results reported below are the test set results.

The results show that for predicting the total percentage of fat with PLSR, normalization according to the total biomass (strategy A) outperforms normalization according to total lipids (strategy B) (see Table 1). This confirms that standardization of the spectra with respect to the total absorbance is equivalent to standardizing the spectra according to the total biomass. This results in peak heights, which give information about the relative concentration of different compounds. When we aim at predicting the total amount of fat, this is favorable.

When establishing calibration models for lipids that are given in percentage with respect to the total amount of lipids, a different strategy is needed. In this case we suggested to normalize spectra according to the total absorbance in the C-H stretching region, 3050–2800 cm${}^{-1}$, which can be considered as approximately proportional to the amount of lipids. We referred to this strategy as strategy B. Following this strategy, we assume that the peak heights after normalization no longer contain information about the total concentration of lipids with respect to the total biomass. This method is more appropriate when analyzing the lipid profiles (see Table 1), since information about the total amount of lipids is expected to be removed from the data. Peaks are normalized such that the peak highs correlate with concentrations of different lipids per total amount of lipids.

### 2.3. Using Constituent Spectra in the EMSC: Analyte and Interferent Spectra

The EMSC model can be extended with the constituent model spectra to explicitly model the known chemical variability. According to the Beer–Lambert law in Equation (9), the EMSC reference spectrum can be expressed in terms of the chemical compounds;

(1) $$Z\left(\tilde{\nu }\right)=\sum_{j=1}^{J}c_{j}k_{j}\left(\tilde{\nu }\right)$$

where $c_{j}$ is the concentration of compound *j*, and $k_{j}$ is the characteristic absorptivity. Therefore, the EMSC model can be written

(2) $$Z_{app}\left(\tilde{\nu }\right)=b\cdot \left\{\sum_{j=1}^{J}c_{j}k_{j}\left(\tilde{\nu }\right)\right\}+a+d\cdot \tilde{\nu }+e\cdot {\tilde{\nu }}^{2}+\epsilon \left(\tilde{\nu }\right).$$

Assuming that all concentrations sum to 1, $\sum c_{j}=1$, we can write

(3) $$\sum_{j=1}^{J}c_{j}k_{j}=\sum_{j=1}^{J}c_{j}\left(k_{j}-Z_{ref}\left(\tilde{\nu }\right)\right)+Z_{ref}\left(\tilde{\nu }\right).$$

This makes it possible to rewrite Equation (2) to

(4) $$Z_{app}\left(\tilde{\nu }\right)=b\cdot Z_{ref}\left(\tilde{\nu }\right)+a+d\cdot \tilde{\nu }+e\cdot {\tilde{\nu }}^{2}+b\cdot \left\{\sum_{j=1}^{J}c_{j}\Delta k_{j}\left(\tilde{\nu }\right)\right\}+\epsilon \left(\tilde{\nu }\right)$$

where $\Delta k_{j}$ is the difference spectrum $\left(k_{j}-Z_{ref}\left(\tilde{\nu }\right)\right)$. To simplify the expression, one can exchange $b\cdot c_{j}$ by $h_{j}$.

(5) $$Z_{app}\left(\tilde{\nu }\right)=b\cdot Z_{ref}\left(\tilde{\nu }\right)+a+d\cdot \tilde{\nu }+e\cdot {\tilde{\nu }}^{2}+\sum_{j=1}^{J}h_{j}\cdot \Delta k_{j}\left(\tilde{\nu }\right)+\epsilon \left(\tilde{\nu }\right)$$

According to Equation (5), constituent spectra are often referred to as difference spectra, since they represent chemically differences between the main constituents and the reference spectrum. Using the difference spectrum and not the complete spectrum of the constituent in the EMSC avoids collinearity between model spectra, which would result in a non-unique solution to the least squares regression (ill-conditioning). Therefore, the constituent spectra do not resemble chemical absorbance spectra of chemical or biological compounds.

We distinguish between analyte constituent spectra and interferent constituent spectra, and they are treated differently in the EMSC. Interferent spectra contain information about unwanted signals which do not relate to the sample, and should therefore be removed from the spectra. Examples include absorbance signals from paraffin in spectra of paraffin embedded pollen samples [10], and bound water in meat tissue sections resulting from day-to-day variations in humidity [15]. By removing the effects of interferents, we reduce the need for calibration data to build reliable models, as well as increase the model interpretability [11]. Analyte spectra, on the other hand, contain information about major chemical variations that we do not want to remove from the spectra. The main purpose of adding analyte spectra is to quantify a known constituent. However, it can also have a stabilizing effect on the EMSC by increasing the modeling capability.

When including constituent spectra in the EMSC, the model reads

(6) $$Z_{app}\left(\tilde{\nu }\right)=a+b\cdot Z_{ref}\left(\tilde{\nu }\right)+f\cdot Z_{ana}\left(\tilde{\nu }\right)+g\cdot Z_{int}\left(\tilde{\nu }\right)+\epsilon \left(\tilde{\nu }\right)$$

where $Z_{ana}$ and $Z_{int}$ represent an analyte difference spectrum and interferent difference spectrum, respectively. An EMSC model can of course include several analyte and interferent spectra, while we select one of each here for simplicity. Note that we replace the parameters $h_{j}$ from Equation (5) by *f* and *g* in order to highlight the difference between analyte and interferent spectra. Polynomial spectra can of course also be added as model spectra. The analyte and interferent spectra are handled differently when the corrected spectrum is calculated;

(7) $$\begin{array}{cc} Z_{corr}\left(\tilde{\nu }\right) & =\frac{Z_{app}-a-g\cdot Z_{int}\left(\tilde{\nu }\right)}{b}\\ \; & =Z_{ref}\left(\tilde{\nu }\right)+\frac{f\cdot Z_{ana}\left(\tilde{\nu }\right)+\epsilon \left(\tilde{\nu }\right)}{b}. \end{array}$$

While the interferent signals are removed from the measured spectrum, the analyte signals are kept. It is now possible to examine the relative content of the constituents by comparing the ratio $\frac{f}{b}$ or $\frac{g}{b}$ among spectra in the dataset.

To see examples of the use of interferent spectra in the EMSC, we refer the reader to [12,16]. Several EMSC-based models are built by including spectra as interferent spectra. Among the most known EMSC-based methods we find the Mie extinction EMSC (ME-EMSC) [13], the fringe EMSC [17] and the replicate EMSC [18,19]. Throughout the remainder of the paper, we illustrate how analyte spectra can be used to gain additional information about the chemical composition of the samples. However, first, we need to discuss the orthogonality between model spectra, as well as some common pitfalls in the use of constituent spectra.

### 2.4. Orthogonality and Pitfalls When Using Constituent Spectra

**Figure 3:**
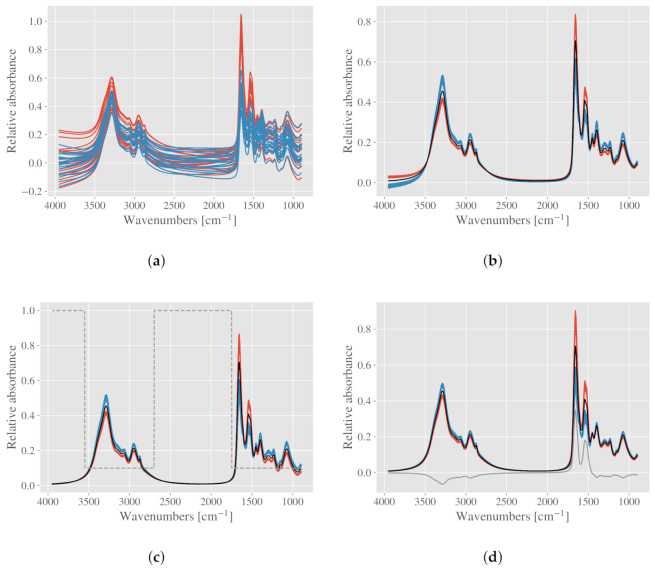
Using EMSC models on a simulated dataset consisting of two chemically different groups. (**a**) Simulated spectra, physical effects are added to the simulated pure absorbance spectra. Spectra corrected by (**b**) EMSC with polynomials up to the 2nd degree; (**c**) EMSC with weights, shown in dashed gray; (**d**) EMSC with an analyte constituent spectrum, shown in gray.

In addition to the parameterization of chemical compounds, adding chemical model spectra can affect the EMSC normalization in a similar way as applying weights. As described above, low weights can be applied to regions with large variability. Alternatively, a known variability could be added to the EMSC model as an analyte spectrum. In the following, we demonstrate this approach using the example of the two simulated groups. In order to highlight some of the advantages of normalization with EMSC compared to for example vector normalization and peak normalization, we introduce physical features to the simulated spectra by adding random constant, linear and quadratic baselines, scaled between −0.1 and 0.1. The disturbed spectra are shown in Figure 3a. Although this is a simulated example, it demonstrates nicely how baseline correction and normalization can be achieved simultaneously with EMSC, whereas other normalization procedures would require baseline correction prior.

We start by correcting the simulated spectra in Figure 3a with a basic EMSC, containing polynomials up to the second degree. This results in the corrected spectra in Figure 3b. It is evident that the chemical variation in the amide I and II peaks affects the correction, and introduces artefacts in the baseline. The artefacts are most clearly seen at 4000 cm${}^{-1}$, where the red and blue group has a systematic difference. This systematic difference can be explained by the fact that due to statistical interference between the chemical difference and the model spectra, some of the important chemical differences are modeled by the polynomial model spectra. These unwanted effects can be avoided by either applying weights, or by adding constituent spectra to the EMSC to explicitly model the chemical variability.

Once again, we need to decide how we want to normalize the spectra. If the goal is to standardize with respect to the total amount of biomass, we can apply the weights shown in Figure 1d and account for the chemical variation in all active spectral regions. The result of the preprocessing by weighted EMSC is shown in Figure 3c, where the artefacts in the baseline are removed. Alternatively, one can add an analyte constituent spectrum, which expresses the chemical difference between the two groups. Clearly, such an approach requires some a priori knowledge. The analyte spectrum used in this study (shown in gray in Figure 3d) is the difference between the means of each group. The resulting corrected spectra are shown in Figure 3d, where we once again see that the artefacts vanish. No weights are used in this correction. As we can see from Figure 3c,d, applying weights can in some cases result in a very similar correction as for including analyte spectra. The main advantage of using EMSC with analyte spectra is that the chemical differences are parameterized and available for the subsequent data analysis.

If one instead wishes to normalize the spectra according to the biomass, but neglect the variation in the amide I and II peaks, this could be achieved by applying zero weights only for the amide I and II region. The spectra will then look like shown in Figure 1a. Alternatively, a constituent spectrum which expresses only the differences in the amide I and II between the groups could also be added. This type of normalization would highlight the differences in protein content, while the rest of the biomass is standardized (results not shown).

The example above is somewhat artificial and serves only the purpose to demonstrate the use of weights and constituent spectra. Later in the paper we show how the same techniques can be applied to real measured data.

#### Orthogonality between Model Spectra

At this point, some comments about orthogonality in the EMSC model should be given. Since the EMSC parameters are found by least squares regression, all model spectra in the EMSC should ideally be orthogonal to each other. The basic model spectra such as the baselines expressed by polynomials are not strictly orthogonal, but linearly independent such that the parameter estimation can be considered as independent. If model spectra such as analyte or interferent spectra are highly dependent on each other, the regression problem for the parameter estimation becomes partially ill-posed, and the parameters cannot be estimated unambiguously. Therefore, all model spectra should ideally be orthogonal to each other.

An interesting situation arises if the basic EMSC model as given in Equation (14) is used for correction of spectra, and subsequently analyte model spectra are then estimated from the loadings of a PCA of the corrected spectra [20]. The advantage of adding the PCA loadings after EMSC corrections is that they are definitely orthogonal to the basic EMSC model spectra that have been used for correcting the data before PCA. However, if the extended EMSC model (extended by PCA loadings as an analyte) is now once more applied to the same data, the correction will be identical for both models. The reason is that when adding a constiuent spectrum (either analyte or interferent) which is orthogonal to all other EMSC model spectra, the constituent spectrum does not affect the parameter estimation for the other model spectra. This can be seen by comparing Equation (14) with Equation (7). If $Z_{ana}$ is orthogonal to the rest of the model spectra in Equation (6) (EMSC with constituents), the only difference from Equation (13) (a regular EMSC) is that the parts of $Z_{app}$, which would otherwise end up in ϵ, is parameterized by $Z_{ana}$ instead. When the constituent spectrum is an analyte spectrum, the correction result is therefore not different for the two EMSC models (with and without orthogonal analyte spectrum), since neither ϵ nor $Z_{ana}$ is removed from $Z_{app}$. On the other hand, if the model spectra that are added to the EMSC model are interferent spectra, i.e., the aim is to remove their contribution to the spectra $Z_{app}$, the correction result will in general be different for both models (EMSC with and without orthogonal interferent spectra). When parts of $Z_{app}$ is parameterized by $Z_{int}$ instead of ϵ, the corrected spectra are affected. This is seen from Equation (7), since $Z_{int}$ is removed from $Z_{app}$.

Here, a note should be given that performing a PCA on corrected spectra is practically the same as performing PCA on residuals of the EMSC models [19]. Therefore, when we in this paper refer to PCA on residuals of an EMSC model, it can be considered equivalent to performing PCA on the spectra corrected by the same EMSC model.

Although orthogonal model spectra are desired, in the example of the two groups where the difference spectrum was added as an analyte constituent spectrum to the EMSC, the difference spectrum was in fact not orthogonal to the rest of the model spectra. The aim in this example was in fact to change the corrected spectra to remove the artefacts in the baseline by expanding the basic EMSC. The model capacity is increased by allowing explicitly modeling the chemical variability. A change in the corrected spectra would not occur if the analyte spectrum was orthogonal to the rest of the model spectra. The use of an orthogonal analyte spectrum would therefore not remove the artifacts in the baseline.

If the aim is to further improve the correction result by adding an analyte spectrum, the analyte spectrum should not be completely orthogonal to the previously used model spectra (if the same previously used model spectra are reused in the new model), even though the least squares regression require independent model spectra, as described above. Therefore, one can either change the previously used model spectra in the new model or assure otherwise that the new model spectrum is not orthogonal and has some collinearity with the other model spectra. However, the main message here is that when adding an orthogonal model spectrum as an analyte spectrum, the user has to be prepared that the correction result is not changed. As long as the degree of collinearity is kept low, ill-conditioning does not pose a big problem in the EMSC. In the following we demonstrate the detrimental effects of a common pit-fall, i.e., to add model spectra with a high degree of collinearity to the EMSC.

**Figure 4:**
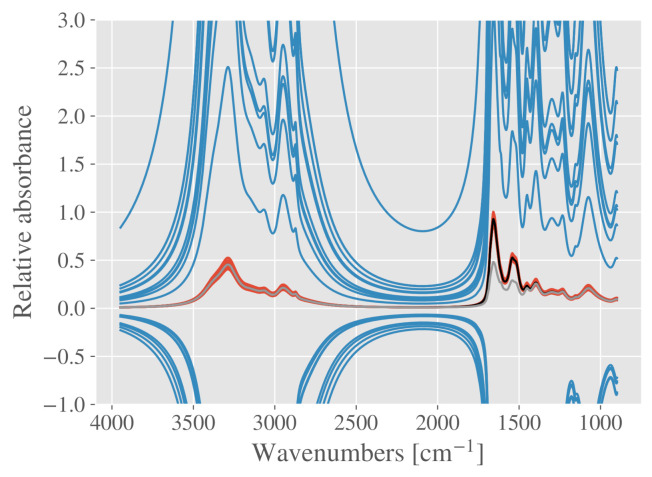
Major pitfall when adding constituent spectra to EMSC models; if the constituent spectra are not orthogonal to the rest of the model spectra, the least squares regression becomes ill-posed, and the parameter estimation becomes highly unstable. The corrected spectra from the blue group demonstrate that the scaling parameter is wrongly estimated. The reference spectrum in the EMSC model (mean of red group) is shown in black, while the analyte constituent spectrum is shown in gray (mean of blue group).

In order to illustrate the problem of dependent analyte spectra, we set up an EMSC model where the mean of the red group from Figure 1a serves as a reference spectrum. The mean of the blue group is then added to the model as an analyte spectrum. Since the mean spectra of the two groups are highly dependent, and one is used as a reference model spectrum, while the other one is used as an analyte spectrum, the estimation of the parameters becomes an ill-posed problem. The resulting corrected spectra are shown in Figure 4, where it is evident that the scaling parameter is correctly estimated for the red group, but severely underestimated for the blue group. It even becomes negative for some spectra of the blue group. To avoid ill-conditioning, constituent spectra should ideally be orthogonal (or close to orthogonal, as described above) to the rest of the model spectra. This is the reason why constituent spectra are also called difference spectra, since they represent chemical differences from the reference spectrum, rather than a spectrum that is highly dependent on the reference spectrum.

### 2.5. Using Constituent Spectra for Estimating Chemical Compounds

In the next sections we give two examples of how analyte spectra can be used to access additional information about the sample. We illustrate in the following sections how EMSC parameters can be used in both a regression task and a segmentation task.

#### 2.5.1. Case Study II: Estimation of Glucose in ATR Spectra of Growth Media

**Figure 5:**
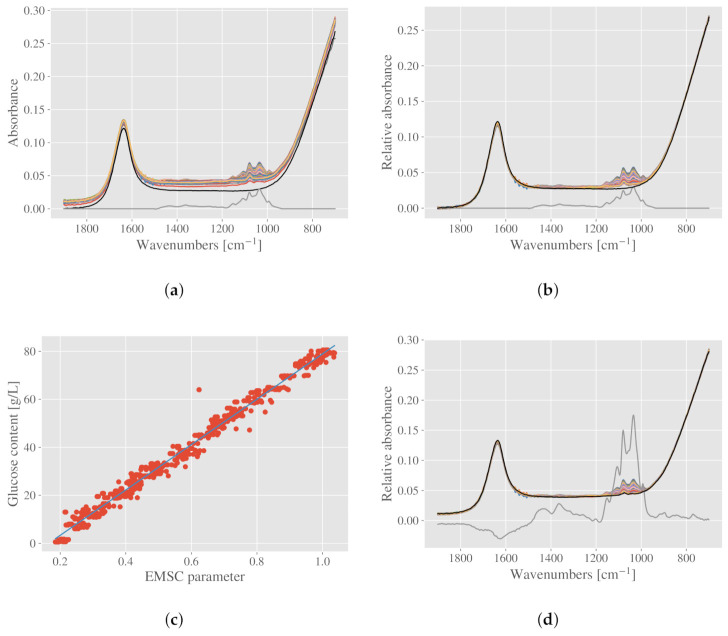
Correcting ATR spectra of growth media by an EMSC with a glucose analyte spectrum. (**a**) Raw spectra of growth media shown together with a pure water spectrum in black and an estimated pure glucose spectrum in gray. (**b**) The corrected spectra are shown together with the pure water spectrum (black), which is used as a reference spectrum in the EMSC model, and the pure glucose spectrum (gray), which is used as an analyte spectrum. (**c**) Regressing the parameter corresponding to the analyte spectrum to the glucose content. An RMSE of 2.81 g/L was achieved, with a corresponding $R^{2}$-score at 0.99. The red dots show glucose content versus the EMSC parameter. The regression line is shown in blue. (**d**) An approximate pure water spectrum can be estimated from the dataset (shown in black) and used as a reference spectrum. Based on a PCA on the residuals, an estimated pure glucose spectrum can be found in the first principal component. The glucose spectrum can be added to the EMSC as an analyte spectrum. The corrected spectra are also shown in the figure.

Measured attenuated total reflectance (ATR) spectra of growth media (growth media dataset described in Section 3.1.2) are shown in Figure 5a, together with the pure water spectrum (black) and estimated pure glucose spectrum (gray). The estimated pure glucose spectrum was found by measuring a water solution with glucose, and subtracting the water signal. For ATR spectra, there are often little signs of baseline variations since scattering does not take place in contact reflection measurements. Therefore, an EMSC with only a constant baseline (MSC) is sufficient for normalizing the spectra [21]. Since an estimated pure glucose spectrum is available, we add this spectrum to the EMSC model as an analyte spectrum, while the pure water spectrum serves as a reference spectrum. The glucose spectrum represents chemical signals we do not want to remove from the measured spectra, and is therefore treated as an analyte spectrum. The corrected spectra are shown in Figure 5b. From the corrected spectra there are different ways of estimating the glucose content. One alternative is to regress the glucose content on the EMSC parameter, which correspond to the glucose analyte spectrum. The EMSC parameter is first divided by the scaling parameter to represent relative content, as described in Section 2.3. The univariate regression results are shown in Figure 5c, and the root mean square error (RMSE) is 2.81 g/L, with a corresponding $R^{2}$-score at 0.99. The glucose content ranged from 0.55 to 80 g/L, with a mean value at 39.4 g/L and standard deviation of 23.2 g/L.

Often, we do not have spectra of pure compounds available. In the following, we show how the same analysis can be performed with only the ATR spectra belonging to the growth media available. Two steps are needed, and we start with a basic EMSC. From the dataset we first select the spectrum that is closest to pure water. This is done by selecting the spectrum with the lowest 1080 cm${}^{-1}$ peak compared to the absorbance at 1200 cm${}^{-1}$. The peak at 1080 cm${}^{-1}$ is clearly related to glucose, as can be seen in the pure glucose spectrum in Figure 5a. The spectrum with the smallest difference between the absorbance at wavenumbers 1080 cm${}^{-1}$ and 1200 cm${}^{-1}$ is expected to represent a water spectrum, and will serve as the reference spectrum in the EMSC. The obtained reference spectrum is shown in black in Figure 5d. Since most of the chemical variation is present in the region 950–1200 cm${}^{-1}$, we put zero weights to this region and apply the EMSC correction. The difference between using weights and not using weights is small in this case. In order to estimate a glucose spectrum, we consider the residuals of the EMSC model. In the residuals we find the unmodelled parts of the spectra, i.e., that part that is not represented by the physical EMSC model spectra and the reference spectrum. By taking a PCA on the residuals of the whole dataset, the first loading resembles a glucose spectrum (see Figure 5d in gray). Alternatively, one could perform the PCA on the EMSC corrected spectra, which leads to a nearly identical result. The obtained spectrum (first loading from the PCA) is added as an analyte constituent spectrum to the EMSC, and we can now perform the new EMSC correction. This time, weights are not needed, as the chemical variability is handled by the constituent spectrum. Finally, the EMSC parameter corresponding to the constituent spectrum can be used in a linear univariate regression with the glucose content. The parameter is first divided by the scaling parameter. An RMSE of 2.89 g/L is achieved, with an $R^{2}$-score at 0.98.

It is once again important to note that when an analyte spectrum is estimated from the residuals, the resulting corrected spectra will not differ from an EMSC without the analyte spectrum. This is because the analyte spectrum is orthogonal to all model spectra, and can therefore not affect the calculation of the other EMSC parameters. Since the analyte spectrum is not removed from the measured spectrum in the correction, the only result from adding the analyte to the model is that information related to the analyte $f\cdot Z_{ana}$ is captured and transferred from ϵ in Equation (7) to the added analyte spectrum. If the goal with adding analyte spectra is to modify the corrected spectra, one must allow some collinearity between the analyte and the model spectra. The degree of correlation should however be kept low.

For comparison, another simple way to estimate the glucose content is to perform a linear univariate regression of the glucose content on the peak value at 1080 cm${}^{-1}$. This resulted in a model with an RMSE at 3.27 g/L and $R^{2}$ at 0.98.

#### 2.5.2. Case Study III: Detection of Connective Tissue and Myofibers in Infrared Microspectroscopy of Beef Loin Sections

**Figure 6:**
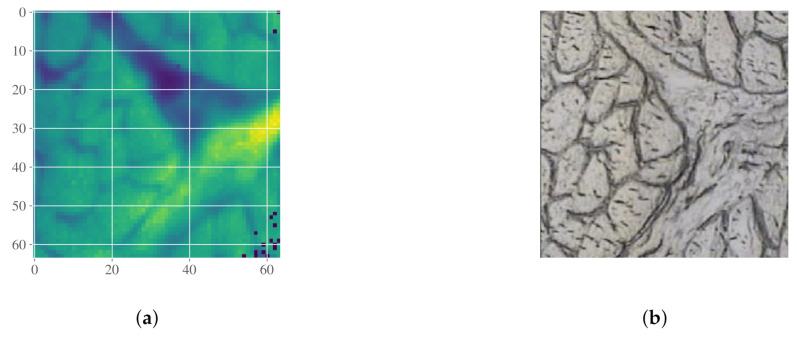

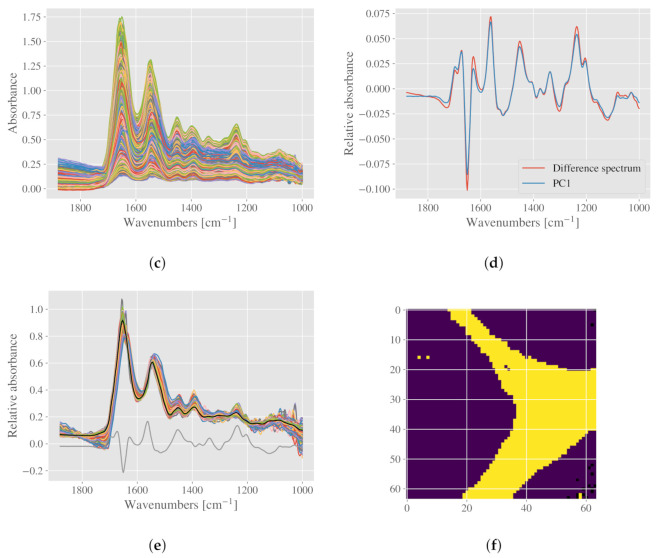
(**a**) Infrared image of beef loin tissue section, at 1653 cm${}^{-1}$. (**b**) Optical microscopy image of the same tissue section. (**c**) Raw spectra from the infrared image. (**d**) Difference spectrum shown in red (difference between mean of myofiber and connective tissue spectra) together with the first principal component from the PCA on the residuals in blue. The principal component is scaled to show that it contains almost exactly the same information as the difference spectrum. (**e**) Corrected spectra shown together with the mean spectrum (black), which is used as a reference in the EMSC, and the first principal component (gray) used as analyte spectrum. (**f**) Mask based on the analyte spectrum parameter. Yellow pixels refer to connective tissue, and blue to myofiber.

In the following example we demonstrate how analyte spectra can be used in a segmentation task for differentiating myofibers from connective tissue in infrared images of sectioned beef loin. The dataset is described in Section 3.1.3. The raw image plotted at the amide I peak at 1653 cm${}^{-1}$ is shown in Figure 6a, and the corresponding optical microscopy image is shown in Figure 6b. The spectra in the image are shown in Figure 6c. An EMSC is performed on the raw spectra, using the mean of the spectra as reference and a constant and linear baseline. The chemical differences between each pixel and the reference are now contained in the residuals. By performing a PCA on the residuals, we structure this variation and make it possible to parameterize the differences from the mean spectrum. The first loading from the PCA is shown in blue in Figure 6d. In the same figure we show the difference between the mean spectrum of a region corresponding to myofiber and a region from connective tissue in red. The regions are found by correlating the visual image to the infrared image. Figure 6d demonstrates that the loading contains information about chemical differences between the two groups. By adding the loading to the EMSC model as an analyte spectrum, we are able to parameterize this difference. The corrected spectra are shown together with the first principal component (used as analyte spectrum) in gray and the mean spectrum (used as reference) in black in Figure 6e. Subsequently, a segmentation can be performed based on the parameter of the constituent spectrum. The result is shown in Figure 6f. A positive parameter indicates that the pixel is connective tissue (yellow), while a negative value indicates myofiber (blue).

Again it is worth mentioning that an analyte spectrum that is estimated from the residuals of the EMSC model did not affect the resulting corrected spectra. We would like to add that the segmentation can also be performed directly on the first principal component scores, which would yield exactly the same results as shown in Figure 6f. In this example, however, the aim is to show how the EMSC parameters relate to concentration of different compounds, and we therefore stay within the EMSC framework.

## 3. Theory and Methods

### 3.1. Datasets

#### 3.1.1. Filamentous Fungi Dataset

A dataset consisting of FTIR spectra from six strains of Mucoromycota oleaginous filamentous fungi was used in this study. The strains were *Amylomyces rouxii* CCM F220, *Mucor circinelloides* VI 04473, *Mucor circinelloides* FRR 5020, *Mucor racemosus* UBOCC A 102007, *Rhizopus stolonifer* CCM F445, and *Umbelopsis vinacea* CCM F539. The fungi were cultivated in duplicates (i.e., two biological replicates) using six different growth conditions, described in [22]. FTIR spectra were collected with a Vertex 70 FTIR spectrometer coupled with a high throughput screening extension (HTS-XT) unit (both Bruker Optik, Ettlingen, Germany). Spectra were recorded in the range 4000–400 cm${}^{-1}$, with spectral resolution of 6 cm${}^{-1}$ and digital spacing of 1.928 cm${}^{-1}$. A total of 64 scans were averaged for each spectrum, and 3 technical replicates were recorded per sample. There were in total 216 spectra in the dataset. The OPUS software (Bruker Optik GmbH, Ettlingen, Germany) was used for data acquisition and instrument control.

Reference analyses for lipid content and fatty acid profiles were performed using a gas chromatography 7820A System (Agilent Technologies, Santa Clara, CA, USA), as described in [23].

#### 3.1.2. Growth Media Dataset

Growth media used for cultivating filamentous fungi were measured with single-reflection attenuated total reflectance (SR-ATR) coupled to a Vertex 70 FTIR spectrometer (Bruker Optik, Ettlingen, Germany). The spectra were recorded with a spectral resolution of 4 cm${}^{-1}$, digital spacing of 1.928 cm${}^{-1}$, and 32 scans, using the horizontal SR-ATR diamond prism with 45 ${}^{\circ }$ angle of incidence on a Specac (Slough, UK) High Temperature Golden Gate ATR Mk II. Spectra of pure water and water solution with glucose were also recorded. From the glucose solution, a pure glucose spectrum was estimated by subtracting the water signals. The OPUS software (Bruker Optik GmbH, Ettlingen, Germany) was used for data acquisition and instrument control. The glucose content of the growth media was measured in g/L with an UltiMate 3000 UHPLC system (Thermo Fisher Scientific, Waltham, MA, USA). For more details about the growth media dataset, see [24].

#### 3.1.3. Beef Loin Dataset

The beef loin dataset refers to an infrared image of a cryo-sectioned beef loin. The sample was collected from a *Longissimus dorsi* muscle from Norwegian Red Cattle, as described in [20]. The tissue section was measured with an IR microscope (IRscope II) coupled to an Equinox 55 FT-IR spectrometer (both Bruker Optik GmbH, Ettlingen, Germany). An image of 64 × 64 pixels was collected, consisting of infrared spectra in the range 3800–900 cm${}^{-1}$. The spectra were recorded using 8 cm${}^{-1}$ spectral resolution. The OPUS software (Bruker Optik GmbH, Germany) was used for data acquisition and instrument control. More details about the experiment can be found in [20].

### 3.2. Baseline and Multiplicative Effects in Infrared Spectroscopy

In the following we give the theoretical background of the extended multiplicative signal correction (EMSC) model. We start by briefly describing baseline and multiplicative effects in infrared spectroscopy. The introduction is kept brief, as this topic has been covered several times elsewhere, see for example [11]. In the following, we describe infrared transmission experiments, while the EMSC model is also applicable to spectra recorded with different methods, as for example attenuated total reflectance (ATR) [21].

Infrared absorbance spectra *Z* are defined through the transmittance *T* as

(8) $$Z\left(\tilde{\nu }\right)=-\log_{10}T(\tilde{\nu })=-\log_{10}\frac{I(\tilde{\nu })}{I_{0}\left(\tilde{\nu }\right)}$$

where $I_{0}$ is the intensity of the incoming radiation, and *I* is the intensity of the radiation which transmits through the sample. $I_{0}$ is measured by removing the sample from the incident beam.

According to the Beer–Lambert law, the absorbance spectrum is proportional to the optical thickness, *d*, of the sample. The absorbance spectrum can be expressed in terms of the concentration $c_{j}$ of each absorbing species *j*;

(9) $$Z\left(\tilde{\nu }\right)\approx \left[\sum_{j=1}^{J}c_{j}k_{j}\left(\tilde{\nu }\right)\right]\cdot d$$

where *J* is the total number of absorbing species and $k_{j}$ is the characteristic absorptivity of each species. Since the absorbance spectrum is scaled proportional to the sample thickness, a parameter which in practice is difficult to control in an infrared experiment, scaling effects are commonly observed in absorbance spectra. Equation (9) represents an ideal case, i.e., when scattering of the incident radiation can be neglected [25,26,27]. In this situation, the absorbance spectra are often called *pure absorbance* spectra.

However, in real-world measurements, scattering and other physical effects can often not be neglected. Scattering leads to loss of radiation at the detector, and therefore affects the absorbance spectrum. Measured spectra that are affected by physical effects are called *apparent absorbance* spectra, or $Z_{app}$. Variations in the intensity of the light source can for example create constant baseline shifts in the absorbance spectra. If the intensity of the source radiation is varied by a factor of α between the two recordings $I_{0}$ and *I*, we can express the absorbance as

(10) $$\begin{array}{cc} Z_{app}\left(\tilde{\nu }\right) & =-\log_{10}\frac{I(\tilde{\nu })}{\alpha I_{0}\left(\tilde{\nu }\right)}\\ \; & =-\log_{10}\frac{I(\tilde{\nu })}{I_{0}\left(\tilde{\nu }\right)}+\log_{10}\alpha \end{array}$$

where the $\log_{10}\alpha$ term accounts for a constant baseline shift. In addition to the scaling effect and constant baseline shift, there are numerous examples of situations where we would expect more sophisticated physical features to be present in the spectra. One example is Mie scattering, which results in scattering features that have been observed in measurements of spherical or approximately spherical samples, such as single cells [13,28,29]. Another example is multiple internal reflections in thin film samples, which causes fringes in the infrared spectra [17,30].

Before infrared absorbance spectra are analyzed, common practice is to first preprocess the spectra to remove unwanted features that do not relate to the chemical composition of the sample. Most multivariate models benefit from preprocessing [2,19]. An extensively used preprocessing technique for infrared spectra is the multiplicative signal correction (MSC) [5]. MSC is a model-based technique, which means that it builds on a physical model, more specifically the Beer–Lambert law from Equation (9) and intensity variations from Equation (10). MSC takes advantage of the fact that infrared spectra share a very similar overall shape. Therefore, each measured spectrum $Z_{app}$ is modeled around a reference spectrum $Z_{ref}$;

(11) $$Z_{app}\left(\tilde{\nu }\right)=a+b\cdot Z_{ref}\left(\tilde{\nu }\right)+\epsilon \left(\tilde{\nu }\right)$$

where the reference spectrum is a pure, scatter-free spectrum, often taken as the mean spectrum from the dataset [6]. The parameter *b* takes the scaling effect into account, while the constant baseline shift from Equation (10) is represented by the constant *a*. The unmodeled part of $Z_{app}$, i.e., the chemical differences between each spectrum and the reference, is contained in the residual term ϵ. The MSC parameters *a* and *b* are determined by least squares regression. In order to standardize the measured spectra, the physical effects are removed by subtracting the constant baseline shift and scaling the spectra according to the reference spectrum;

(12) $$Z_{corr}\left(\tilde{\nu }\right)=\frac{Z_{app}\left(\tilde{\nu }\right)-a}{b}.$$

$Z_{corr}$ is a scatter-free spectrum that is normalized according to the reference spectrum.

In addition to the scaling effect and the constant baseline shift, it is common to observe signals of diffuse scattering in the absorbance spectra. Diffuse scattering leads to broad features in the baseline, where the offset does not change significantly from one wavenumber to the next. These broad features are often well corrected by extending the MSC model, adding polynomial terms, called polynomial model spectra, to Equation (11). In its most basic form, the extended MSC (EMSC) reads

(13) $$Z_{app}\left(\tilde{\nu }\right)=a+b\cdot Z_{ref}\left(\tilde{\nu }\right)+c\dot{\tilde{}}\nu +d\cdot {\tilde{\nu }}^{2}+\epsilon \left(\tilde{\nu }\right),$$

where the corrected spectrum is found by removing the physical features from the measured spectrum:

(14) $$Z_{corr}\left(\tilde{\nu }\right)=\frac{Z_{app}\left(\tilde{\nu }\right)-a-c\cdot \tilde{\nu }-d\cdot {\tilde{\nu }}^{2}}{b}.$$

The polynomial model spectra are centered around the midpoint of the wavenumber region, such that the polynomials are either symmetric or anti-symmetric around the midpoint. This is described in greater detail in the Supplementary Material. Here, the term EMSC will also cover MSC models, stating the orders of the polynomials used.

It is possible to extend the model by polynomials of order higher than two, however, one should be careful not to include too high orders when correcting FTIR spectra. Higher order polynomial spectra could start modeling broad chemical features, such as the broad O-H stretching region around 3500–2600 cm${}^{-1}$. For FTIR spectra recorded in the range 4000–500 cm${}^{-1}$, second order polynomials and below are typically used. One should keep in mind that if cutting the spectra, for example selecting only the fingerprint region 1800–500 cm${}^{-1}$, one should consider to limit the polynomials to the first degree only. For Raman spectra, however, the chemical features in the spectrum are of very sharp nature, and therefore polynomials of higher orders are frequently used. In [12] it is reported that polynomials up to the seventh degree do not pose any over-fitting problems in Raman spectra.

One of the major advantages of the EMSC is the possibility to add spectra that represent chemical features to the model. This enables explicit modeling of known chemical variations in the dataset, which in turn can provide valuable information to the data analysis. We call these chemical model spectra *constituent spectra*. The use of constituent spectra are discussed in detail in Section 2, where we also show common pitfalls.

## 4. Conclusions

In this paper we have clarified some of the common misunderstandings about EMSC preprocessing when the EMSC model contains constituent spectra, and we have shown how to avoid major pitfalls when using such models. The importance of using orthogonal model spectra has been discussed, and examples of how to construct them were given. Further, the effect of weighting in the EMSC was discussed, showing that weighting affects the normalization. The optimal normalization strategy depends on the study. Alternatively to applying weights, we have shown that analyte constituent spectra can be used. Analyte spectra allows modeling of known compounds, and provides additional information about the dataset. Examples of how to utilize the additional information were given, including a regression task and a segmentation task.

## Figures and Tables

**Table 1:** Results from PLSR on the spectra shown in Figure 2. Models for predicting total % fat of the total biomass, as well as % PUFA of the total amount of fat was established. Every instance in the table starts with ( $A_{opt}$), which was found based on the full cross-validation on the training set. For the test set, the % fat range from 19.7 to 87.1, with an average of 41.4 and standard deviation at 16.0. The % PUFA values range from 8.68 to 41.3, with an average of 23.5 and standard deviation at 6.64.

Normalization Strategy	Predicting % Fat of Total Biomass	Predicting % PUFA of Total Fat
Strategy A	(2) $R^{2}=0.89$, RMSE = 5.24	(4) $R^{2}=0.67$, RMSE = 3.79
Strategy B	(7) $R^{2}=0.69$, RMSE = 8.87	(2) $R^{2}=0.87$, RMSE = 2.37

## Data Availability

The fungi dataset presented in this study is available in the supplementary material in Ref. [22]. The growth media dataset presented in this study is available in the supplementary material in Ref. [24]. The beef loin dataset is available on request from the corresponding author.

## Supplementary Materials

The following are available online at https://www.mdpi.com/article/10.3390/molecules27061900/s1, Figure S1: MSC correction of spectra from the filamentous fungi dataset. All models use polynomi als up to the second degree, and the polynomial model spectra are shown in pink, red and blue in all figures. The reference spectrum is shown in black. (a) Corrected spectra using an EMSC on the full spectral range. (b) Corrected spectra using the region 1800–400 cm^−1^. (c) Corrected spectra using the full spectral range, and applying zero weights in the region 4000–1800 cm^−1^. The weights are shown in dashed grey. Figure S2. EMSC correction of different spectral regions. All models use polynomials up to the first degree, and the polynomial model spectra are shown in pink and red, while the reference spectrum is shown in black. (a) EMSC corrected spectra from the lipid regions. Zero weights are applied to the silent region 2800–1765 cm^−1^. (b) EMSC corrected spectra from the lipid regions. The silent region 2800–1765 cm^−1^ is removed from the spectra before the EMSC. (c) The region 3050–2800 cm^−1^ is corrected separately. (d) The region 1765–1727 cm^−1^ is corrected separately. (e) The corrected spectra from (c) and (d) shown together.

## Sample Availability

Samples of the compounds are not available from the authors.

## Abbreviations

The following abbreviations are used in this manuscript:

EMSC	Extended multiplicative signal correction
FTIR	Fourier transform infrared
HTS-XT	High throughput screening extension
MSC	Multiplicative signal correction
PLSR	Partial least squares regression
PUFA	Polyunsaturated fatty acid
RMSE	Root mean square error
SR-ATR	Single-reflection attenuated total reflectance
