# Supplementary material for: The Use of Constituent Spectra and Weighting in Extended Multiplicative Signal Correction in Infrared Spectroscopy
Source: Molecules. 2022 Mar 15;27(6):1900. doi: 10.3390/molecules27061900 (PMC8948808; doi:10.3390/molecules27061900)
Supplement: Supplementary file 1 [file molecules-27-01900-s001.zip › molecules-1605485-supplementary.pdf]

Supplementary Materials: The use of constituent spectra and weighting in Extended multiplicative signal correction in infrared spectroscopy

Johanne Heitmann Solheim [1]*, Boris Zimmermann [1], Valeria Tafintseva [1], Simona Dzurendová [1], Volha Shapaval [1] and Achim Kohler [1]

1. The effect of cutting spectral regions or applying weights on EMSC polynomial model spectra

The extended multiplicative signal correction (EMSC) builds on the multiplicative signal correction (MSC), and is extended by including model spectra. In the basic EMSC model, polynomial model spectra of 1st and 2nd degree are used to model diffuse scattering. The polynomials are centered around the midpoint of the wavenumber region, such that they are either symmetric or anti-symmetric around the midpoint. Examples of polynomial spectra are shown in Fig. 1(a) (plotted in pink, red and blue). The figure also shows the EMSC corrected filamentous fungi FTIR spectra, with the reference spectrum (mean spectrum) in black. The spectral region covers 4000-400 cm^{-1}.

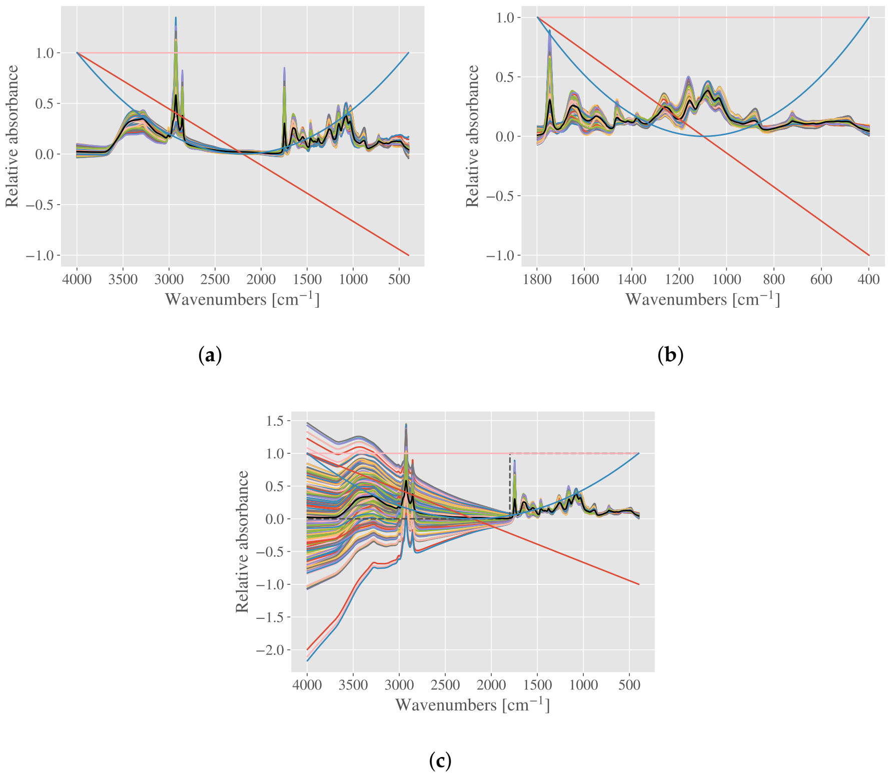

Figure S1. EMSC correction of spectra from the filamentous fungi dataset. All models use polynomials up to the second degree, and the polynomial model spectra are shown in pink, red and blue in all figures. The reference spectrum is shown in black. (**a**) Corrected spectra using an EMSC on the full spectral range. (**b**) Corrected spectra using the region 1800-400 cm^{-1}. (**c**) Corrected spectra using the full spectral range, and applying zero weights in the region 4000-1800 cm^{-1}. The weights are shown in dashed grey.

If the spectral region is cut prior to the correction, the polynomial spectra change accordingly. An example is shown in Fig. 1(b), where the spectra of filamentous fungi are

cut such that the region 1800-400 cm^{-1} is kept. The polynomial spectra are centered around the new spectral midpoint.

Instead of removing a spectral region, one can assign the region more or less importance in the EMSC. Weighting by zero is nearly the same as removing the region completely. The only difference is the construction of the polynomial spectra, as can be seen in Fig. 1(**c**). Here the weights are shown in dashed grey. The corrected spectra are in the region 1800-400 cm^{-1} approximately equal to the cut corrected spectra in Fig. 1(**b**). Therefore, weighting by zero is approximately the same as removing a spectral region completely.

In the following we look closer at normalization according to the lipid content, as discussed in case study I in the article. To normalize according to the lipids, one could select the region 3050-1727 cm^{-1} and perform a weighted EMSC where the region 2800-1765 cm^{-1} is assigned zero weights. This approach is shown in 2(**a**). The polynomial model spectra are also plotted, and the reference is shown in black. Weights are shown in dashed grey. As expected, the down weighted region has a large variability.

An alternative approach is to remove the region 2800-1765 cm^{-1} prior to the correction. We see from Fig 2(**b**) that the polynomial spectra are the same as before. Therefore, the EMSC corrected spectra are equal in the spectral regions 3050-2800 cm^{-1} and 1765-1727 cm^{-1}.

The two approaches are done in order to normalize according to the lipid content. However, since the two lipid regions are so narrow and so far apart, the corrected spectra are still affected by strong baseline variations. In order to remove the baseline variations, the spectral regions should be preprocessed separately. This corrected spectra and the EMSC model spectra are shown in Fig. 2(**c**) and Fig. 2(**d**). The regions are shown together in Fig. 2(**e**). When the spectra are normalized according to lipids, the ester peak loose most of its predicting value, as described in case study I.

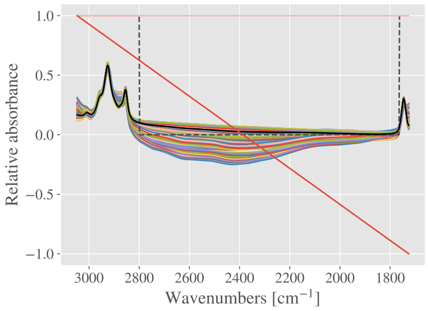

(**a**)

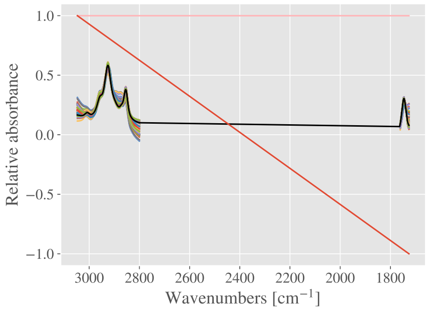

(**b**)

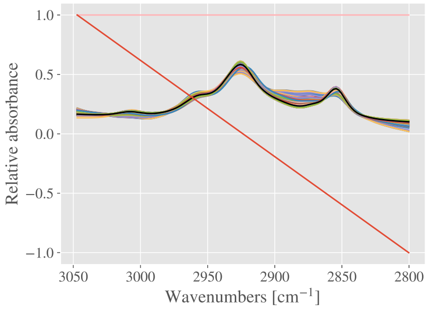

(**c**)

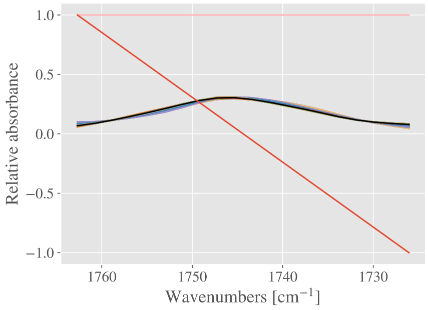

(**d**)

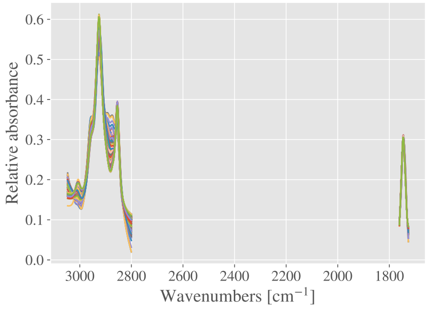

(**e**)

Figure S2. EMSC correction of different spectral regions. All models use polynomials up to the first degree, and the polynomial model spectra are shown in pink and red , while the reference spectrum is shown in black. (**a**) EMSC corrected spectra from the lipid regions. Zero weights are applied to the silent region 2800-1765 cm^{-1}. (**b**) EMSC corrected spectra from the lipid regions. The silent region 2800-1765 cm^{-1} is removed from the spectra before the EMSC. (**c**) The region 3050-2800 cm^{-1} is corrected separately. (**d**) The region 1765-1727 cm^{-1} is corrected separately. (**e**) The corrected spectra from (c) and (d) shown together.
